# Glucose dysregulation and glycemic phenotyping in chronic migraine

**DOI:** 10.3389/fneur.2025.1719724

**Published:** 2026-01-14

**Authors:** Christina A. Nelson, Kyle W. Reavely, Matthew R. Jennings, Brandon J. Burger, Alexander C. Kim, David W. Sant, Kyle B. Bills

**Affiliations:** Noorda College of Osteopathic Medicine, Provo, UT, United States

**Keywords:** chronic migraine, glucose metabolism, glucose tolerance test, hyperglycemia, hypoglycemia, insulin, phenotype

## Abstract

**Background:**

Emerging evidence suggests that a metabolic mismatch between cerebral energy demand and supply may be a contributing factor to the onset of migraine. Studies have drawn connections between migraine and conditions such as hypoglycemia, fasting, GLUT1 transporter deficiency, insulin resistance, and diabetes, highlighting the role of metabolic dysregulation in migraine susceptibility. Understanding these metabolic patterns could pave the way for personalized migraine treatments targeting glucose regulation.

**Methods:**

We conducted a retrospective cohort analysis of chronic migraine subjects (>15 headache days/month) using continuous glucose monitoring (CGM; *n* = 131) and oral glucose tolerance tests (GTTs; *n* = 247). Continuous glucose monitoring data were analyzed from a prospective cohort of 24 healthy controls using metrics such as inter/intraday standard deviation (SD), average daily risk range (ADRR), mean amplitude of glycemic excursion (MAGE), mean glucose excursion (MGE), mean of daily differences (MODDs), continuous overall net glycemic action (CONGA24), post-prandial glucose recovery time (PGRT), and post-prandial area under the curve (PP-AUC). Subjects were clustered into three groups based on OGTT responses using K-means clustering to identify possible postprandial phenotypes.

**Results:**

Compared to age- and gender-matched standards of normal GTT response, individuals with chronic migraine had significantly lower glucose values at fasting and 2 h. CGM data further revealed that migraine subjects exhibited greater glucose variability, including increased day-to-day (MODDs) and within-day (CONGA24) glycemic variability, and higher interday and intraday standard deviation during waking hours. Post-prandial dysregulation was also observed in measures of MAGE, MGE, PGRT, and PPAUC, with all metrics except PGRT differing significantly from controls during waking hours. Clustering of glucose tolerance test results identified three distinct phenotypes, each characterized by unique glucose and insulin response profiles.

**Discussion:**

Chronic migraine subjects exhibited postprandial glucose dysregulation and greater glycemic variability than healthy controls. The identified GTT phenotypes reveal distinct glucose regulation patterns, suggesting the existence of different migraine-associated metabolic profiles. Future research will investigate factors contributing to these phenotypes and their implications for migraine pathogenesis. These findings support the potential for developing targeted migraine treatments informed by glucose dysregulation patterns.

## 1 Introduction

Migraine affects an estimated 12% of the population in the United States, with approximately 2.5% of individuals with episodic migraine progressing to chronic migraine annually (1). Chronic migraine is a particularly debilitating subset of migraine, defined by the Headache Classification Committee of the International Headache Society as a headache occurring on more than 15 days per month for over 3 months, with at least 8 days exhibiting features of migraine with or without aura (2).

Treatment of those diagnosed with chronic migraine is both complex and difficult, involving a combination of lifestyle modifications, traditional migraine pharmacologic agents, and novel treatments, including a variety of biologics targeting calcitonin gene-related peptide (3). Future therapeutics rely on understanding the pathophysiology underlying the progression from episodic to chronic migraine, which has not yet been fully elucidated. Several risk factors, however, have been identified, including obesity, acute migraine medication overuse, ineffective treatment of episodic migraine, and female sex (4).

The relationship between migraine and hypoglycemia was first described almost 90 years ago, but the hypothesis of extracranial vasodilation dominated most research in the subsequent decades (5, 6). It was not until the 1980s that the relationship between cerebral metabolic supply and demand in the context of migraine was further explored by Amery (7). A relatively newer branch of migraine research centers on the “neuroenergetic” hypothesis, which describes migraine as the result of brain energy deficits, with a subset postulating that insulin resistance in the brain may underlie the transition from episodic to chronic migraine (8). Recent studies supporting an insulin link suggest a relationship between migraine attacks and abnormal mitochondrial metabolism of glucose and lipids (9).

One line of study has relied on genome-wide association studies to discover genes associated with biological pathways that could be implicated in both migraine and glucose traits. These studies have found candidate risk loci for both diseases, but the results are inconsistent, indicating that further investigation is necessary (10). The epidemiological association between diabetes and migraine has been explored in a meta-analysis of cross-sectional studies that revealed a positive association between a history of migraine, particularly without aura, and the occurrence of diabetes mellitus (11). The implication of glucose metabolism in migraine is further supported by the influence of dietary factors, specifically fasting and mild reactive hypoglycemia, on migraine induction (12).

Instead of a direct connection between diabetes and migraine, insulin receptor regulation may link the pathophysiology of these two diseases. This role of insulin sensitivity has been explored, with the severity and impact of migraine attacks being higher in patients with insulin resistance than in those without (13). In addition, insulin levels have been found to be higher in migraineurs (not necessarily chronic) when compared to both healthy controls and those experiencing non-migraine headaches (14). Another possible target for further research is GLUT1, which is responsible for the transport of glucose across the blood–brain barrier. A series of case reports describe patients with GLUT-1 deficiency caused by SLC2A1 mutations, whose presenting symptoms include hemiplegic migraine (15–17).

With the association between glucose traits and migraine being established in the literature, further exploration of glucose dysregulation, specifically glycemic variability and post-prandial imbalance, in those suffering from chronic migraine could provide beneficial insight into the metabolic processes underlying the propagation of this debilitating disease (18). Understanding these metabolic patterns, specifically in the context of chronification, could also pave the way for novel interventions, including dietary modifications, glucose stabilization strategies, or pharmacological approaches aimed at enhancing an individual's cerebral energy homeostasis. We hypothesize that altered glycemic variability and impaired post-prandial glucose regulation contribute to the progression from episodic to chronic migraine, and that characterization of these metabolic disturbances will highlight potential therapeutic targets.

## 2 Methods

### 2.1 Participants

Data were collected as part of a retrospective cohort analysis at an outpatient migraine rehabilitation clinic in Utah, USA, with a prospective control group recruited for comparison. Migraine subjects were aged between 18 and 75 years, with a median age of 32, and comprised 87% women and 13% men). Control subjects (*n* = 24) were recruited from the Noorda College of Osteopathic Medicine community and selected from an age- and sex-matched population using frequency matching to approximate the distribution of the migraine cohort. The median age was 28.3 years, with 72% women and 28% men. Continuous glucose monitoring data were collected from a retrospective cohort analysis of 131 chronic migraine subjects and a prospective analysis of 24 matched controls. Oral glucose tolerance test (OGTT) data were collected from intake exams in a retrospective cohort analysis of 247 chronic migraine subjects with a control group selected from a normative dataset consisting of an age- and sex-matched population using frequency matching of age and sex to approximate the distribution of the migraine population at the cohort level from the CDC National Center for Health Statistics National Health and Nutrition Examination Survey from 2015 to 2016 (547 individuals; 86% women and 13% men with a median age of 37.5 years). Not all migraine subjects with OGTTs had CGMs, and vice versa; some migraine subjects were represented in both study groups, but not all. Control groups were fully separated with CGM data from the 24 controls recruited from Utah and OGTT data from the normative dataset. Chronic migraine was defined as >8 migraine days and >15 headache days per month according to ICHD-3 criteria (2). Individuals with diabetes and prediabetes, post-concussive syndrome, epileptic seizures, or drug-use frequency suggestive of medication overuse headache were excluded. Individuals with and without aura were included in the study; 64% of subjects included in the study reported regular occurrence of aura.

### 2.2 Ethical considerations

This study was reviewed and approved by the institutional review board at Noorda College of Osteopathic Medicine, NCOMIRB#22-008N. The authors followed the STROBE recommendations and guidelines. All subjects gave written informed consent for retrospective/prospective analysis. All protected health information (PHI) was removed from electronic data before consolidation and analysis. The coded master list with PHI was kept in a separate password-protected, encrypted file to protect the confidentiality of the research subjects. A power analysis was performed before the study to determine appropriate sample sizes. We reached statistical significance before reaching this threshold and, therefore, stopped recruiting (prospective and retrospective) at this point according to ethical guidelines.

### 2.3 Data collection and analysis

The custom Python functions were built from validated calculations from the publications listed under their respective subject headings and from the GitHub repository: CGMquantify (19).

#### 2.3.1 Average daily risk range (ADRR) and low/high blood glucose indices (LBGI/HBGI)

We computed the Average daily risk range (ADRR) for each participant using a custom Python function. This function quantifies the sum of risky low index (LBGI) and risky high index (HBGI) values observed in a given day to provide a metric for glucose excursion into hypoglycemic and hyperglycemic risk zones (20). First, glucose values were log-transformed and scaled using the formula where G represents the glucose value:


$$\begin{array}{l} f\left(G\right)\;=\;\left(\ln{\left(G\right)}^{1.084}\right)-5.381 \end{array}$$

Low-risk and high-risk scores were then computed using the standard Kovatchev risk function (21, 22):


$$\begin{array}{l} r\left(G\right)\;=\;22.77\;f({G)}^{2} \end{array}$$

Applied to values with *f* (*G*) < 0 for LBGI and *f* (*G*) > 0 for HBGI. The constant 22.77 is algebraically equivalent to the original formulation 10 × 1.509^2^, in which the 1.509 multiplier appears inside *f* (G). We use this equivalent parameterization for consistency with contemporary implementations. Daily low-risk and high-risk scores were defined as the maximum LBGI and HBGI values per day, and ADRR was calculated as the mean of their sum across days.

#### 2.3.2 Mean amplitude of glycemic excursion (MAGE)

We computed mean amplitude of glycemic excursion (MAGE) using a custom Python function. With this function, local minima and maxima were detected and classified by their amplitude. Only excursions with a magnitude of ≥20 mg/dl were considered significant and included in the MAGE calculation. The MAGE score was then computed as the mean of all significant excursions within the dataset, where *N* represents the number of significant glucose excursions (23):


$$\begin{array}{l} MAGE\;=\;\frac{1}{N}\overset{n}{\underset{i\;=\;1}{\sum }}|Peak_{i}-Valley_{i}| \end{array}$$

#### 2.3.3 Mean glycemic excursion (MGE)

We calculated mean glycemic excursion (MGE) using a custom Python function. This function calculates the mean glucose level of values that fall outside the upper and lower thresholds. For this function, we defined the lower threshold as μ – σ, and the upper threshold as μ + σ. Glucose values outside of thresholds were considered significant and included in the MGE calculation. The MGE score was calculated as the mean of out-of-threshold values as follows, where *N* is the number of out-of-threshold glucose values:


$$\begin{array}{l} MGE\;=\;\frac{1}{N}\overset{N}{\underset{i\;=\;1}{\sum }}G_{i},\;G_{i}\notin \left[\mu -\sigma ,\;\mu +\sigma \right] \end{array}$$

#### 2.3.4 Mean of daily differences (MODDs)

To evaluate intra-day glucose variability, we used a custom Python function to measure the mean of daily differences (MODDs)s, which measures the daily fluctuations in glucose levels at the same time on consecutive days. We used the following formula, where *G*_*i, t*_ represents the glucose value at a given time *t* on day *i*, and *G*_*i, t*−24*h*_ is the corresponding value recorded at the same time as the previous day.


$$\begin{array}{l} MODD\;=\;\frac{1}{N}\overset{n}{\underset{i\;=\;1}{\sum }}|G_{i,t}-G_{i,t-24h}| \end{array}$$

The mean of these absolute differences across all valid time points is then computed to calculate the MODD score.

#### 2.3.5 Continuous overall net glycemic action (CONGA24)

Continuous overall net glycemic action (CONGA24) is similar to MODDs, but instead of focusing on absolute values, it emphasizes the variability of fluctuations. We calculated CONGA24 using a custom Python function. Glucose values are compared between 24 h and coded based on the standard deviation of the differences. We used the following formula, where *G*_*i, t*_represents the glucose value at a given time *t* on day *i*, and *G*_*i, t*−24*h*_ is the corresponding value recorded at the same time as the previous day.


$$\begin{array}{l} CONGA24\;=\;\sigma \left(\left|G_{i,t}-G_{i,t-24h}\right|\right) \end{array}$$

This measure reflects how much variation exists in glucose fluctuations over a full day, emphasizing the spread of differences over their magnitude (MODDs).

#### 2.3.6 Post-prandial glucose recovery time (PGRT)

To assess post-prandial glucose regulation, we calculated the amount of time it took, on average, for glucose levels to return to half of baseline after a peak of ≥40 mg/dl within 60 min (12 glucose readings). The baseline glucose level was determined as the median glucose level within 120 min preceding the detected peak. If glucose did not reach the *T* $\frac{1}{2}$ target in 240 min, the peak was classified as an impaired response, and a maximum recovery time of 240 min was assigned. To prevent double-counting of peaks, a minimum separation of 90 min was enforced before detecting a new peak.

#### 2.3.7 Post-prandial area under the curve (PP-AUC)

To assess the extent of glucose dips below baseline following a postprandial peak, we measured the post-prandial area under the curve (PP-AUC). The postprandial peak was defined as ≥40 mg/dl within 60 min (12 glucose readings). Baseline was established as the median glucose level in the 60 min preceding the detected peak. After a peak, glucose values were measured for 180 min according to the following formula.


$$\begin{array}{r} PP-AUC\;=\;\int_{t_{0}}^{t_{f}}\left(Baseline\;Glucose-Glucose\;\left(t\right)\right)dt,\\ where\;Glucose\;\left(t\right)\;<\;Baseline \end{array}$$

Average PP-AUC was then computed as the mean AUC across all detected peaks.

### 2.4 K-Means clustering

We applied K-means clustering using the sklearn.cluster module in Python to a set of subjects with both glucose and insulin values for full OGTTs. The clustering was based on extracted glucose features from the OGTTs. The extracted features were fasting, 1 h, 2 h, and 3h glucose and insulin, and glucose and insulin AUC. K-means clustering was performed with *k* = 3 clusters. *k* = 3, 4, 5, 6 were also tested, but three clusters showed the most reliable and distinct borders. This clustering was performed on 64 migraine subjects who had both glucose and insulin data for OGTTs.

### 2.5 Statistical analysis

For each measure, waking hours were defined as 09:00 to 23:59, and sleep was defined as 00:00 to 06:00. The hours of 06:00 to 09:00 were excluded due to variability in waking times and timing of subjects' first meals. Migraine subjects were compared to controls using Student's *t*-tests. Comparisons between the OGTT glucose and insulin responses between phenotypes were performed using a one-way ANOVA. Welch's *t*-tests and ANOVAs were performed in Excel using XLMiner Analysis ToolPak. Adjustments for multiple comparisons (false discovery rate) were included for the continuous glucose monitor tests. Analysis of normality was conducted using the Shapiro–Wilk test using the scipy.stats module in Python.

## 3 Results

### 3.1 Oral glucose tolerance and hourly glucose averages in chronic migraine

**Figure 1:**
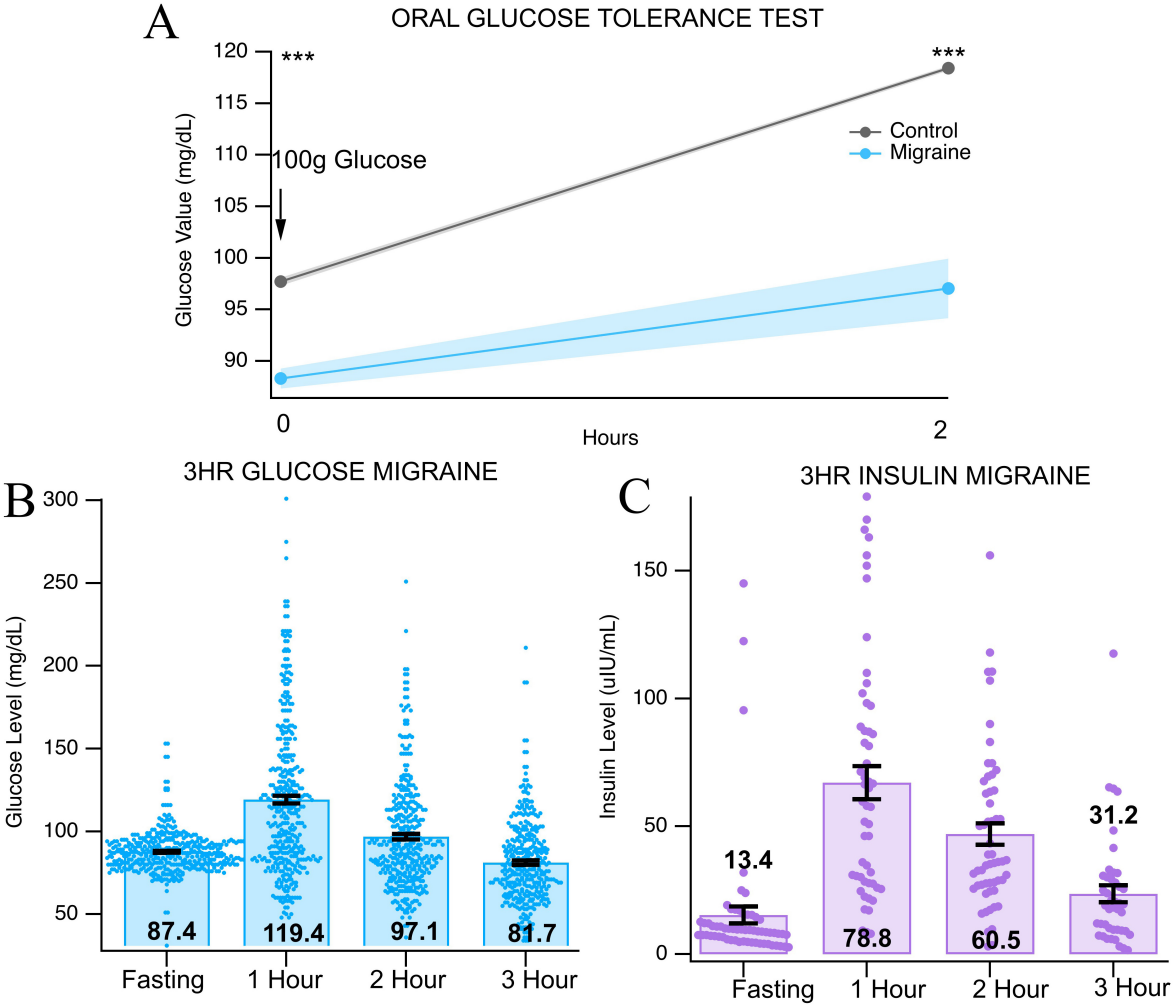
Glucose tolerance averages and SD. **(A)** Oral glucose tolerance test results from chronic migraine subjects compared to normative dataset. **(B, C)** results from 100 g glucose challenge **(B)** glucose values and **(C)** insulin values. *** indicate *p* < 0.001.

Evidence suggests that reduced blood glucose levels are associated with migraine attacks (24–26). To investigate further, OGTTs from 247 subjects with chronic migraine were evaluated to characterize glucose and insulin levels. The results were compared against an age-matched and sex-matched normative dataset (*n* = 547). Fasting glucose for migraine subjects (87.4 ± 0.77 mg/dl) was significantly lower than the normative dataset [97.69 ± 0.39 mg/dl; *t*_(713)_ = −10.46, *p* < 0.0001, Figure 1A]. At 2 h, glucose was also significantly lower for migraine subjects (97.11 ± 2.25 mg/dl) than the normative group [118.36 ± 0.22 mg/dl; *t*_(714)_ = −13.88, *p* < 0.0001, Figure 1A]. Note that the normative dataset did not have data points for 1 and 3 h. Figures 1B, C shows glucose and insulin values for all 3 h (76 subjects had insulin data fasting-3 h). Serum insulin levels at fasting and 3-h were 13.4 ± 1.69 and 31.2 ± 3.55, respectively, both higher than accepted norms.

### 3.2 Insights from continuous glucose monitoring

**Figure 2:**
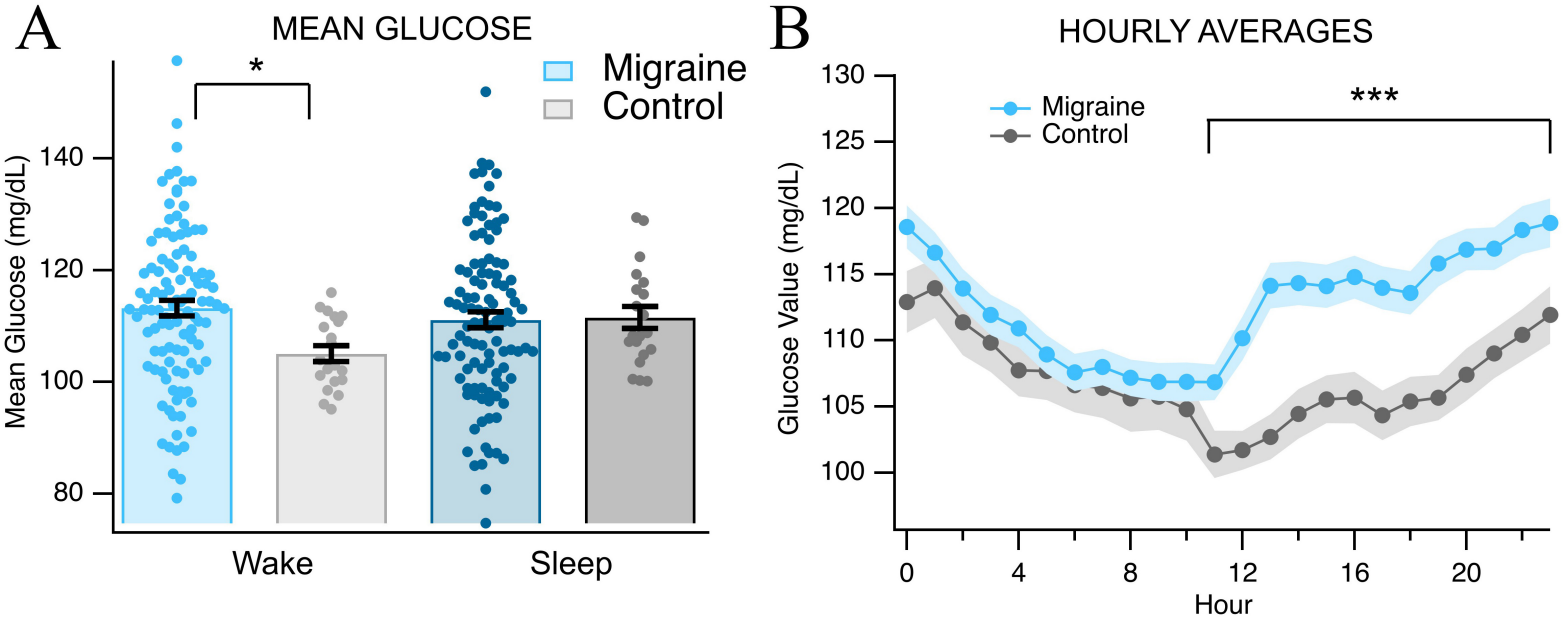
Glucose averages. Data from migraine subjects from continuous glucose monitoring compared to control subjects. **(A)** Wake (10 a.m.−12 a.m.) vs. sleep (12 a.m.−10 a.m.) data. **(B)** Comparison of hourly averages across 24 h. Asterisks * and *** indicate *p* > 0.05 and *p* < 0.001, respectively.

**Figure 3:**
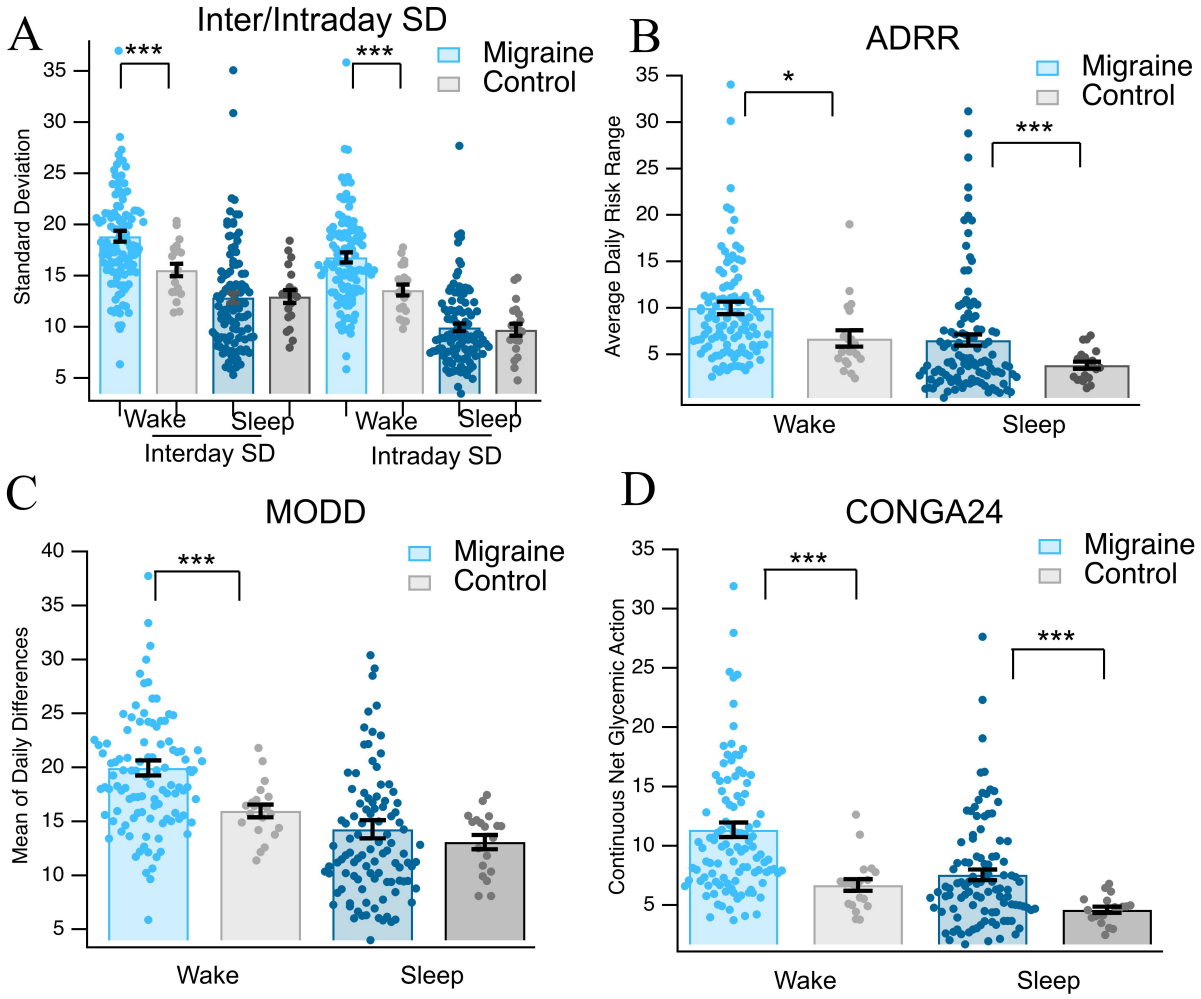
Glycemic variability. **(A)** Mean glucose values from CGM for migraine and controls. **(B)** Average daily risk range scores for migraine and controls. **(C)** Mean of daily differences for migraine and controls. **(D)** Continuous net glycemic action for migraine and controls. Asterisks * and *** indicate *p* < 0.05 and *p* < 0.001, respectively.

We next evaluated hourly glucose averages based on continuous glucose monitoring (*n* = 131 migraine) compared to non-migraine, age-matched and sex-matched controls (*n* = 24 control). Migraine subjects showed no significant differences during sleeping hours (00:00–6:00) but significant differences between average hourly glucose during waking hours [09:00–23:59; *t*_(100)_ = 0.65, *p* = 0.257, *q* = 0.314; *t*_(118)_ = 3.52, *p* < 0.001, *q* < 0.001, Figures 2A, B]. There were significant differences in overall glucose values and inter- and intraday standard deviations during waking hours between migraine subjects and controls [interday *t*_(52)_ = 3.67, *p* < 0.001, *q* < 0.001; intraday *t*_(49_) = 3.66, *p* < 0.001, *q* < 0.001, Figure 3A]. There were no significant differences in interday and intraday SD during sleep hours [interday *t*_(44)_ = −0.52, *p* = 0.30, *q* = 0.33; intraday *t*_(34)_ = −0.028, *p* = 0.49, *q* = 0.49, Figure 3A]. As indicated by the increased interday and intraday standard deviations of glucose values and the increased glucose averages during waking hours, there appears to be an emerging phenotype of increased glycemic variability in migraine subjects. It has been suggested that glucose variability (27) could be a key driving factor in the development and maintenance of chronic migraine. As such, we used the data from the CGMs and analyzed three different measures of glycemic variability: average daily risk range (ADRR), mean of daily differences (MODDs), and continuous net glycemic action (CONGA24). Migraine subjects showed greater ADRR scores during both waking [*t*_(44)_ = 2.14, *p* = 0.019, *q* = 0.026, Figure 3B] and sleeping [*t*_(122)_ = 3.53, *p* < 0.001, Figure 3B] hours, suggesting increased incidence of risky high and low glycemic values. Furthermore, during waking hours, migraine subjects had greater MODD values [*t*_(101)_ = 4.37, *p* < 0.001, *q* < 0.001, Figure 3C], indicating instability in glycemic patterns across consecutive days. Additionally, the migraine group had higher CONGA24 scores during sleeping and waking hours, indicating that glucose levels fluctuated more frequently and with greater magnitude than the controls [*t*_(91)_ = 6.43, *p* < 0.001, *q* < 0.001; *t*_(107)_ = 5.19, *p* < 0.001, *q* < 0.001, respectively, Figure 3D]. It is especially interesting to note that while sleeping, and therefore fasting, individuals with migraine have reduced glycemic control and a higher incidence of risky high and low glucose levels, suggesting that this effect cannot be attributed solely to unique patterns of food consumption and diet, but instead, at least partially to general glucose dysregulation.

### 3.3 Post-prandial dysregulation

**Figure 4:**
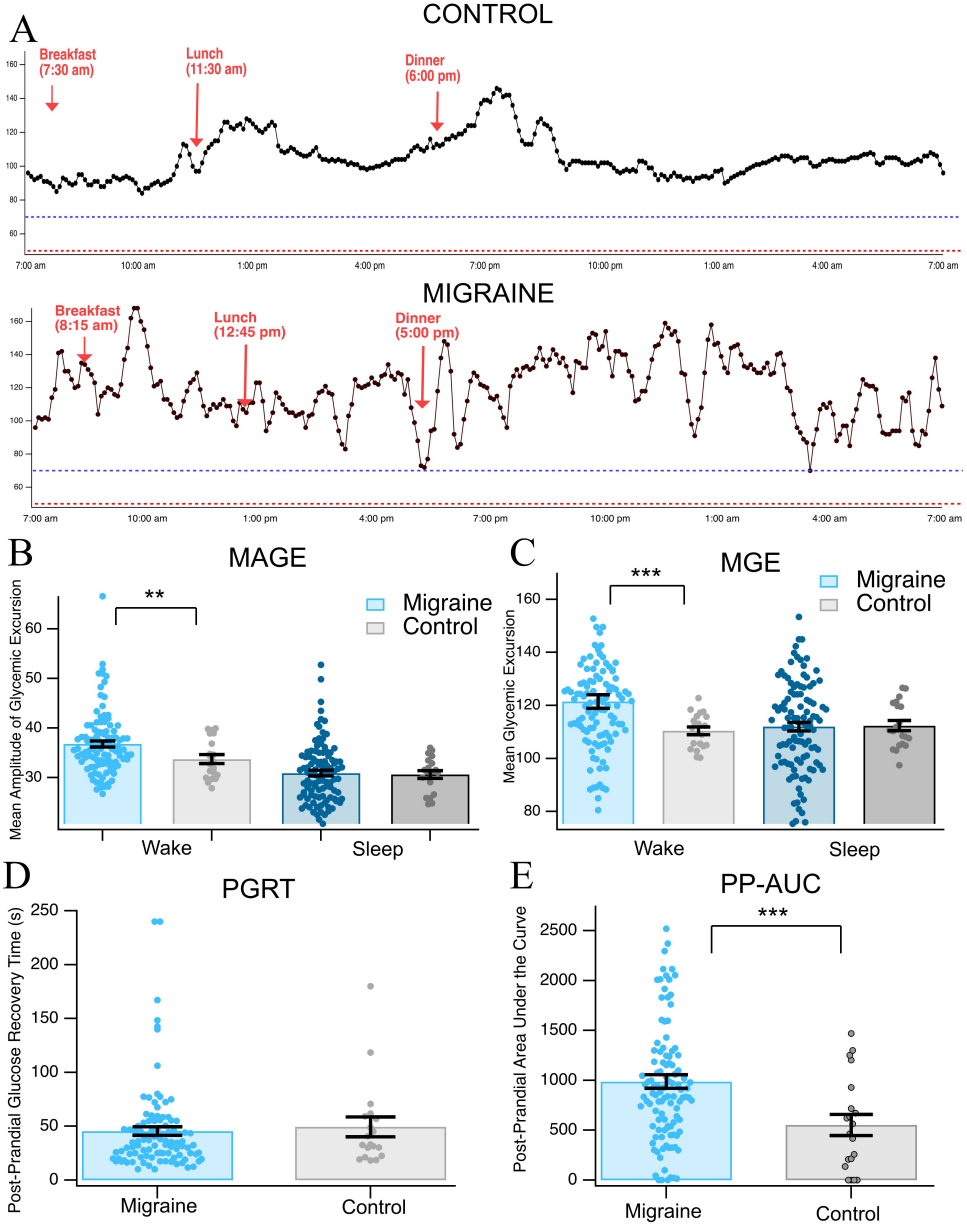
Post-prandial dysregulation. **(A)** Representative trace from CGM of chronic migraine and control, **(B)** mean amplitude of glycemic excursion, **(C)** mean glycemic excursion, **(D)** post-prandial glucose recovery time (1/2 of baseline), and **(E)** post-prandial area under the curve. Asterisks ** and ***, indicate *p* < 0.01 and *p* < 0.001, respectively.

Given the post-prandial abnormalities in the chronic migraine group (Figure 1), we expanded on our investigation of post-prandial glucose regulation. Figure 4A provides representative traces for the trends we found in post-prandial hypoglycemia and glycemic variability in the migraine subjects and a corresponding representative control group (*n* = 131 migraine, *n* = 24 control). We first used the metrics mean amplitude of glycemic excursions (MAGEs), mean glycemic excursions (MGEs), and post-prandial area under the curve (PGRT) to characterize post-prandial events to measure the mean amplitude and absolute value of glucose excursions after a meal. We found that during waking hours, the migraine subjects had greater MAGE and MGE, indicating larger amplitude peaks in response to a meal [*t*_(39)_ = 2.46, *p* = 0.009, *q* < 0.0012; *t*_(114)_ = 3.73, *p* < 0.001, *q* < 0.001, Figures 4B, C, respectively]. We did not find significant differences in PGRT between migraine and control subjects during waking hours [*t*_(25)_ = −0.391, *p* = 0.35, *q* = 0.35, Figure 4D]. Although the time it took for the glucose value to return to half of baseline (PGRT) did not vary between groups, we did see significant differences in the PP-AUC. Those with migraine had 44% more area under the curve than controls, indicating more severe or prolonged dips in glucose below baseline after a meal [*t*_(37)_ = 3.46, *p* < 0.001, *q* = 0.0012, Figure 4E].

### 3.4 Normality testing

**Figure 5:**
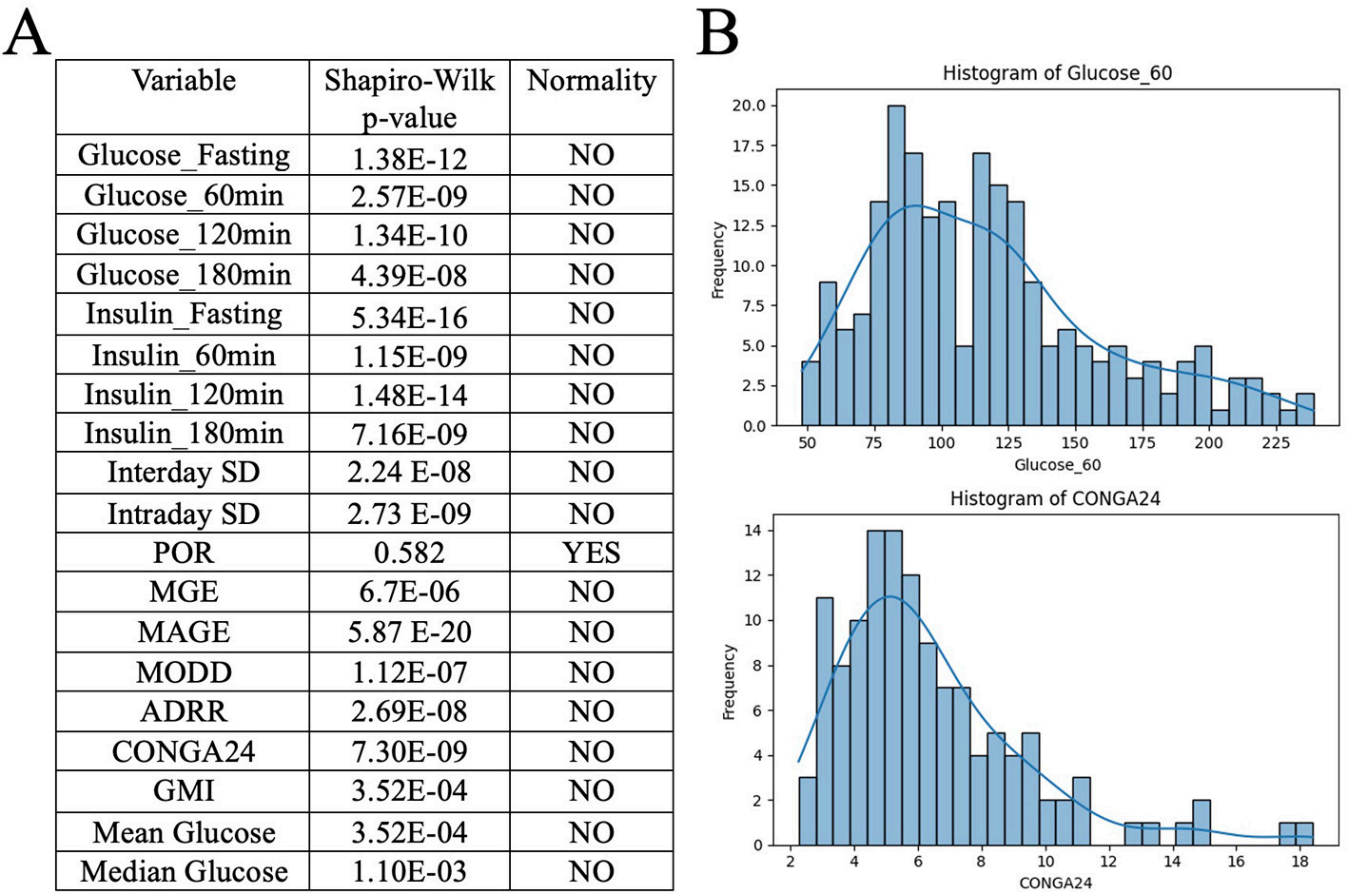
Normality. **(A)** Results from the Shapiro–Wilks test of normality. All tests were not normally distributed except POR. **(B)** Histograms for Glucose_60min and CONGA24 as representative examples.

Due to the nature of the datasets and their resulting graphs, we suspected non-normal distributions. We used a Shapiro–Wilk test of normality for all OGTT and CGM results, found in Figure 4. All tests were not normally distributed, except POR (Figure 5A). Representative histograms are also shown in Figure 5B for Glucose_60min and CONGA24; however, all tests showed the same right skew.

### 3.5 Post-prandial phenotypes in chronic migraine

**Figure 6:**
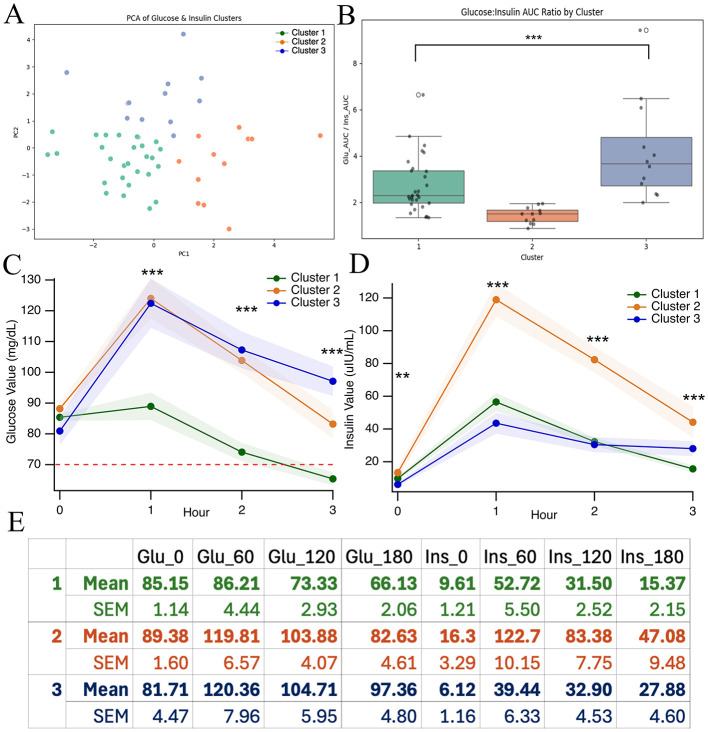
Glucose and insulin phenotypes. Clusters and their associated colors and numbers are kept consistent for this presentation based on this projection. **(A)** 2D PCA projection from analysis of OGTT results, including AUC and glucose: insulin ratios. **(B)** Glucose AUC: Insulin AUC values by cluster. **(C)** OGTT glucose averages by cluster **(D)** OGTT insulin averages by cluster. **(E)** Summary of means and SEMs for each cluster at each time point. Asterisks *** indicate *p* < 0.001.

Based on the non-normal distribution of the OGTT and CGM results (see 3.4), we decided to investigate possible clusters in the dataset (*n* = 64 migraine subjects with both glucose and insulin OGTT data). To identify possible phenotypes in glucose dysregulation, we used a K-means clustering of the OGTT responses. After testing 3–6 clusters, three clusters were chosen because the model had the most distinct and clear borders of all the models (Figure 6A). This model was run repetitively >10 times to ensure reliability, and clustering results came back the same every time. We then plotted the OGTT values based on the cluster given by the K-means model. Figure 6B shows glucose AUC: insulin AUC ratios plotted by cluster, another indication of strong cluster separation. For each hour, we performed a one-way ANOVA comparing glucose values each hour within each group and found that the clusters differed significantly at each time point except fasting [Fasting *F*_(2, 60)_ = 2.37, *p* = 0.1024; 1 h *F*_(2, 60)_ = 12.65, *p* < 0.001; 2 h *F*_(2, 60)_ = 23.15, *p* < 0.001; and 3 h *F*_(2, 60)_ = 21.78, *p* < 0.001, Figure 6C]. These tests were repeated for insulin values showing significant differences between groups at all time points [fasting *F*_(2, 54)_ = 5.61, *p* = 0.0061; 1 h *F*_(2, 58)_ = 30.4, *p* < 0.001; 2 h *F*_(2, 60)_ = 38.20, *p* < 0.001; and 3 h *F*_(2, 58)_ = 10.40 *p* < 0.001, Figure 6D]. Figure 6E gives a table of mean and SEM values for each cluster at each time point, fasting to 3 h, for glucose and insulin.

Data from patient intakes, including comorbidities, medications, demographics, and results from neurocognitive testing, were amassed and compared between clusters. Table 1 shows the results of this investigation with red-marked values that deviate from those of the other clusters. Percentages listed for treatment modalities represent both the frequency of use in the cluster and the percentage that reported some efficacy with the intervention. Of note, cluster 1 shows the highest triptan use at 57% compared to cluster 2 (13.3%) and cluster 3 (25%). Cluster 1 also has significantly lower A1c values than the other clusters (*F*_(2, 36)_ = 3.42, *p* = 0.044). Cluster 2 shows the highest CGRP use at 40% compared to cluster 1 (23.3%) and cluster 3 (16.7%). Cluster 3 shows very high SNRI use at 41.7% whereas both cluster one and cluster 2 use less than 7% (3.3 and 6.7, respectively). Cluster 3 also showed the highest rates of depression and sleep disorder at 33.3% and 16.7%. Rates of neurocognitive deficit, cluster C disorders (anxiety and OCD), and thyroid disorder were similar across clusters. Furthermore, age, gender, and aura appear to be similar across clusters as well.

**Table 1:** Cluster demographics: results from an investigation of comorbidities, demographics, and medications.

**Measure**	**Cluster number**		
	**1**	**2**	**3**
Age	37.1 ± 1.70	38.1 ± 3.40	37.8 ± 4.8
Gender	75% F 25% M	73% F 27% M	92% F 8% M
A1C	5.11±0.05	5.27 ± 0.04	5.29 ± 0.09
Aura	68.9%	53.8%	70.0%
BMI	24.9	26.9	25.4
Triptan use	57.0	13.3%	25.0%
CGRP use	23.3%	40.0%	16.7%
SSRI/SNRI use	26.6%/3.3%	13.3%/6.7%	16.7%/41.7%
TCA use	10.0%	13.3%	8.3%
Propranolol use	10.0%	6.7%	16.7%
Anticonvulsant use	23.3%	20.0%	33.3%
Muscle relaxer/botox	13.3%/20.0%	6.7%/6.7%	16.7%/8.3%
Hypertension	3.3%	7.1%	16.7%
OCD or anxiety	22.5%	28.6%	33.3%
Depression	3.3%	14.3%	33.3%
Sleep disorder	0%	7.1%	16.6%
Thyroid disorder	10.0%	0%	8.3%
Neurocognitive scores	45.9 ±1.20%	46.7 ±1.70%	48.5 ±1.74%

Red-highlighted values are marked to indicate abnormal deviations from other clusters.

## 4 Discussion and future directions

Chronic migraine subjects paradoxically report migraine activity associated with feeding and fasting. Despite the opposing nature of the reports, they are consistent with previous findings suggesting migraine is a disorder that includes neuroenergetic pathology. Our data represent one of the first large-scale studies to explore this hypothesis from the perspective of continuous glucose monitoring, oral glucose tolerance testing, and phenotypical clustering, and further support this hypothesis by demonstrating that glucose dysregulation is a common feature of chronic migraine. Understanding the individual glycemic phenotype of chronic migraine subjects could prove to be an important aspect of diagnostic triaging.

To better understand how systemic glucose regulation varies in chronic migraine, we analyzed OGTT data. Our data demonstrated differential insulin responses over the course of 3 h in response to a 100 g glucose challenge. With all chronic migraine data aggregated, both fasting and 3-h insulin levels, which should both represent a fasting baseline, were elevated from generally accepted values (28–30), while glucose levels were broadly normal at those time points (Figure 1). Conversely, at 1- and 2-h, insulin release was relatively normal while glucose values were significantly lower than expected. We propose that these data suggest a state where hepatic insulin resistance causes elevations in fasting insulin resistance through increased pancreatic β-cell tone with exaggerated peripheral insulin sensitivity post glucose load.

To further evaluate glucose dysregulation and its real-world impact on regular feeding cycles and to examine mechanistic causes, we analyzed continuous glucose monitoring data for sleep/wake patterns and measures of glycemic variability. Chronic migraine subjects demonstrated significantly higher than average blood glucose levels than controls during waking hours (10 a.m.−12 a.m. vs. 12 a.m.−10 a.m.; *p* < 0.0001; Figure 2) and significant differences in interday and intraday standard deviations. Further ADRR and CONGA24 scores were significantly different between chronic migraine and control in both wake and sleep (Figure 3). These findings demonstrate that the increased amplitude and frequency of glucose fluctuation are present in chronic migraine. This is suggestive of dynamic glucose instability rather than simply hyperglycemia or hypoglycemia. Significant differences were also present in the post-prandial glucose variability as measured by MAGE, MGE, and PP-AUC. These metrics demonstrate that chronic migraine subjects experience greater amplitude of excursions and greater overall hypoglycemic dips following meals (Figure 4). Notably, previous studies have demonstrated a connection between hypoglycemia and increased susceptibility to cortical spreading depression (CSD) (31) and compromise of cerebral vasculature following CSD with hyperglycemia (32). This complex interaction between glycemia, CSD, and by extension, migraine onset is relevant considering the currently reported data.

Finally, subjects were clustered according to responses to OGTTs to identify unique phenotypes of glucose dysregulation. With our limited data, a three-cluster model was most reproducible and illuminating. Three distinct phenotypes emerged, and they were cross-analyzed for clinical presentation. Cluster one demonstrated clear reactive hypoglycemia with high fasting insulin and apparent insulin hypersensitivity post glucose load. This cluster also used and reported greater success with triptan use when compared to other clusters. This could possibly point to hepatic insulin resistance with peripheral insulin hypersensitivity, which would mechanistically align with triptan efficacy due to possible regulation of neural response to hypoglycemic dips. Cluster 2 had a broadly normal glucose response with elevated insulin at all time points, suggesting systemic insulin resistance. This cluster reported the highest use and efficacy of CGRP monoclonal antibody medications and less efficacy from triptans. This could be due to more systemic insulin resistance, producing greater hypothalamic neuroenergetic stress and persistently elevated CGRP tone, explaining why a triptan's interruption of a single acute CGRP release elicited by a hypoglycemic would be less effective in this cluster. Cluster 3 had the least pronounced metabolic issues but the highest rate of comorbidities, including higher rates of depression, anxiety/OCD, and sleep disorders. They also reported significantly higher use of SNRI medication. These characteristics could suggest that patients with this phenotype may respond to interventions that target affective and arousal centers in the brain, including SNRIs, behavioral interventions, and sleep stabilizers.

The hybrid retrospective and prospective design of this study introduces potential confounding and bias in the data, resulting in missing information on headache and migraine duration and severity during glucose measurements. We recommend that future studies employ a prospective randomized design. Furthermore, this analysis distinguishes between chronic migraine patients and those with standalone or concurrent medication overuse headaches (MOHs). Future studies could include an analysis of any differences between chronic migraine and those with MOH. Additionally, a normative dataset was used for comparison against the retrospective OGTT group. Although the normative dataset was age-matched and sex-matched to closely resemble the retrospective group, we anticipate that the dataset includes unanticipated features that might introduce bias to the analysis. As such, we recommend that the findings be considered preliminary and a springboard for future studies.

**Figure 7:**
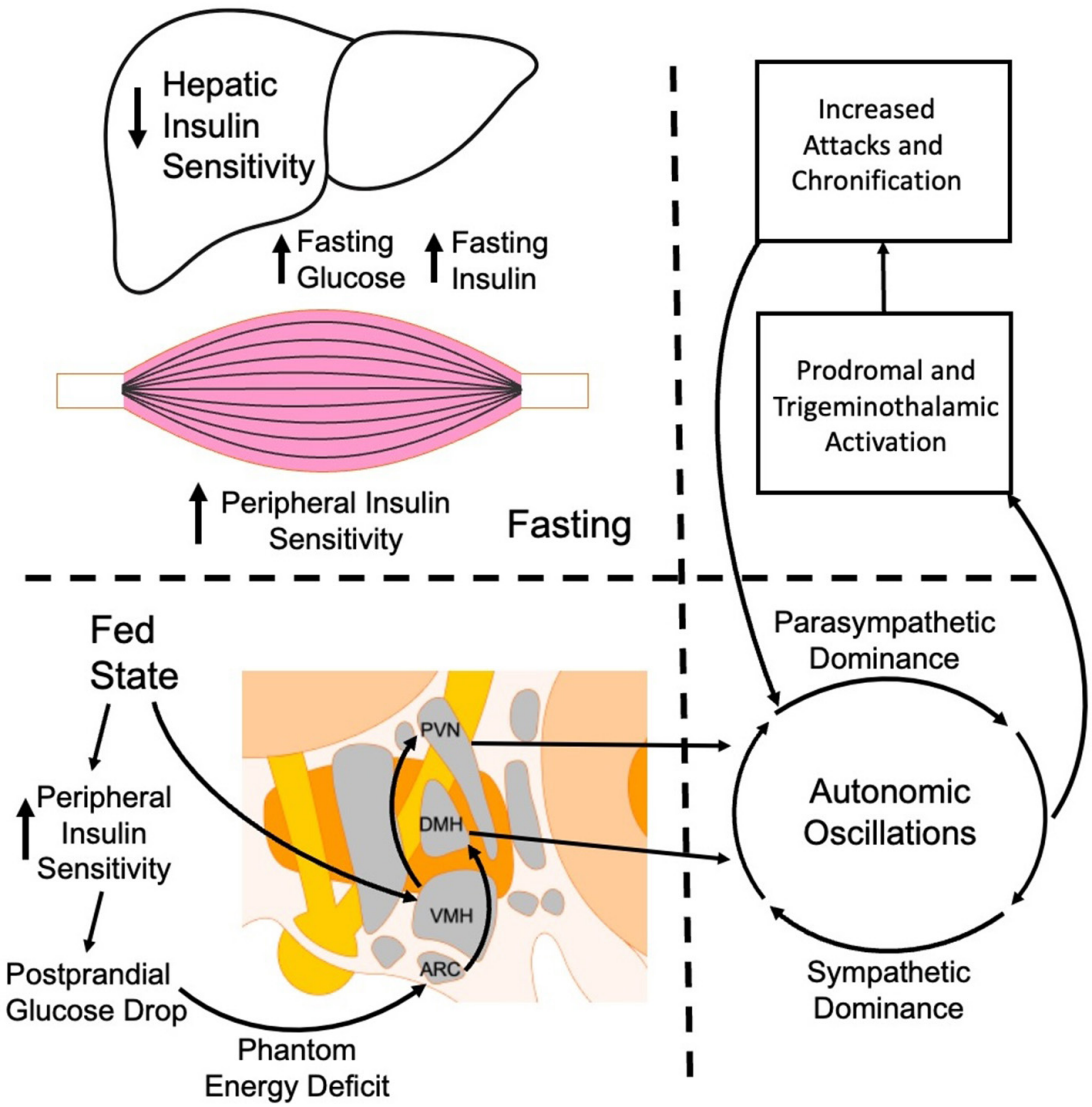
Proposed mechanism of glucose dysregulation leading to migraine chronification.

To explain the role of glucose dysregulation in migraine chronification, we propose the following as a potential theoretical mechanistic framework for scrutiny in future studies (Figure 7).

1. In chronic migraine, post-prandial glucose response is blunted due to paradoxical hepatic insulin resistance with simultaneous peripheral insulin sensitivity. Following a glucose load, enhanced peripheral clearance produces a shallow rise in glucose or a reactive hypoglycemic drop. This blunted glucose rise alters the excitation patterns of glucose-excitatory (GE) and glucose-inhibitory (GI) neurons in the ventromedial (VMH) and arcuate (ARC) hypothalamus. Premature firing of GI neurons signals reduced fuel availability in spite of being in a fed state, eliciting a “phantom energy deficit.”
2. The paradoxical energy state is signaled through the paraventricular nucleus (PVN) to the superior salivatory nucleus, then to the sphenopalatine ganglion to drive parasympathetic outflow. This occurs simultaneously with the normal dorsomedial hypothalamus (DMH) activation of sympathetic tone through the nucleus tractus solitarius (NTS), locus coeruleus (LC), and the intermediolateral column (IML). This produces oscillatory sympathetic/parasympathetic output, which manifests itself in prodromal features.
3. Parasympathetic activation encourages meningeal vasodilation while sympathetic swings contribute to destabilized cardiovascular tone. Trigeminal nociceptors to the dura become hyperexcitable and release CGRP, substance P, and PACAP, which amplify vasodilation and contribute to increased signaling to the trigeminal nucleus caudalis (TNC). Cortical spreading depression is more easily triggered due to relative reductions in glucose availability combined with neuronal volatility when the attack becomes self-propagating.
4. Repeated cycles of hypothalamic dysfunction, autonomic oscillations, and trigeminovascular activation produce chronic neuroinflammatory changes. Chronic cytokine release in the TNC and cortex contributes to central sensitization, contributing to interictal hypersensitivity. Persistent autonomic dysregulation lowers the interictal migraine threshold as maladaptation in VMH and ARC occurs. Together, these changes produce a self-reinforcing loop contributing to migraine chronification.

These findings can provide a theoretical framework for future studies and the potential for more concrete studies exploring the potential for customized treatments based on glucose phenotypes in chronic migraine.

## Data Availability

The raw data supporting the conclusions of this article will be made available by the authors, without undue reservation.

## References

[1] Burch RC Buse DC Lipton RB. Migraine: epidemiology, burden, and comorbidity. Neurol Clin. (2019) 37:631–49. doi: 10.1016/j.ncl.2019.06.001 31563224

[2] Headache Classification Committee of the International Headache Society (IHS) The International Classification of Headache Disorders 3rd edition. Cephalalgia. (2018) 38:1–211. doi: 10.1177/0333102417738202 29368949

[3] Kung D Rodriguez G Evans R. Chronic migraine: diagnosis and management. Neurol Clin. (2023) 41:141–59. doi: 10.1016/j.ncl.2022.05.005 36400552

[4] May A Schulte LH. Chronic migraine: risk factors, mechanisms and treatment. Nat Rev Neurol. (2016) 12:455–64. doi: 10.1038/nrneurol.2016.93 27389092

[5] Blau JN. Migraine pathogenesis: the neural hypothesis reexamined. J Neurol Neurosurg Psychiatry. (1984) 47:437–42. doi: 10.1136/jnnp.47.5.437 6376712 PMC1027817

[6] Gray PA Burtness HI. Hypoglycemic headache. Endocrinology. (1935) 19:549–60. doi: 10.1210/endo-19-5-549

[7] Amery WK. Brain hypoxia: the turning-point in the genesis of the migraine attack? Cephalalgia. (1982) 2:83–109. doi: 10.1046/j.1468-2982.1982.0202083.x 6751554

[8] Del Moro L Rota E Pirovano E Rainero I. Migraine, brain glucose metabolism and the “neuroenergetic” hypothesis: a scoping review. J Pain. (2022) 23:1294–317. doi: 10.1016/j.jpain.2022.02.006 35296423

[9] Gross EC Lisicki M Fischer D Sandor PS Schoenen J. The metabolic face of migraine - from pathophysiology to treatment. Nat Rev Neurol. (2019) 15:627–43. doi: 10.1038/s41582-019-0255-4 31586135

[10] Islam MR Nyholt DR. Glucose-related traits and risk of migraine-a potential mechanism and treatment consideration. Genes. (2022) 13:730. doi: 10.3390/genes13050730 35627115 PMC9141901

[11] Ha WS Nguyen VK Chu MK. Epidemiological linkage between migraine and diabetes mellitus: a systematic review and meta-analysis. J Headache Pain. (2024) 25:158. doi: 10.1186/s10194-024-01868-2 39333866 PMC11438040

[12] Hufnagl KN Peroutka SJ. Glucose regulation in headache: implications for dietary management. Expert Rev Neurother. (2002) 2:311–7. doi: 10.1586/14737175.2.3.311 19810862

[13] Ali M Hussein M Magdy R Khamis A Al-Azayem SA Othman AM . The potential impact of insulin resistance and metabolic syndrome on migraine headache characteristics. BMC Neurol. (2022) 22:422. doi: 10.1186/s12883-022-02966-x 36368970 PMC9652792

[14] Cavestro C Rosatello A Micca G Ravotto M Marino MP Asteggiano G . Insulin metabolism is altered in migraineurs: a new pathogenic mechanism for migraine? Headache. (2007) 47:1436–42. doi: 10.1111/j.1526-4610.2007.00719.x 18052953

[15] Grech O Sassani M Terwindt G Lavery GG Mollan SP Sinclair AJ. Alterations in metabolic flux in migraine and the translational relevance. J Headache Pain. (2022) 23:127. doi: 10.1186/s10194-022-01494-w 36175833 PMC9523955

[16] Horckmans S Van Paesschen W. GLUT-1 transporter deficiency presenting as hemiplegic migraine in an adult. Acta Neurol Belg. (2024) 124:699–700. doi: 10.1007/s13760-023-02387-8 37733158

[17] Scoppola C Magli G Conti M Fadda M Luzzu GM Simula DM . CACNA1A-linked hemiplegic migraine in GLUT 1 deficiency syndrome: a case report. Front Neurol. (2021) 12:679354. doi: 10.3389/fneur.2021.679354 34135856 PMC8200771

[18] Suh S Kim JH. Glycemic variability: how do we measure it and why is it important? Diabetes Metab J. (2015) 39:273–82. doi: 10.4093/dmj.2015.39.4.273 26301188 PMC4543190

[19] Bent B Henriquez M Dunn J. Cgmquantify: python and R software packages for comprehensive analysis of interstitial glucose and glycemic variability from continuous glucose monitor data. IEEE Open J Eng Med Biol. (2021) 2:263–6. doi: 10.1109/OJEMB.2021.3105816 35402978 PMC8901031

[20] Patton SR Clements MA. Average daily risk range as a measure for clinical research and routine care. J Diabetes Sci Technol. (2013) 7:1370–5. doi: 10.1177/193229681300700529 24124966 PMC3876383

[21] Kovatchev BP Cox DJ Gonder-Frederick LA Young-Hyman D Schlundt D Clarke W. Assessment of risk for severe hypoglycemia among adults with IDDM: validation of the low blood glucose index. Diabetes Care. (1998) 21:1870–5. doi: 10.2337/diacare.21.11.1870 9802735

[22] Kovatchev BP Otto E Cox D Gonder-Frederick L Clarke W. Evaluation of a new measure of blood glucose variability in diabetes. Diabetes Care. (2006) 29:2433–8. doi: 10.2337/dc06-1085 17065680

[23] Service FJ Molnar GD Rosevear JW Ackerman E Gatewood LC Taylor WF. Mean amplitude of glycemic excursions, a measure of diabetic instability. Diabetes. (1970) 19:644–55. doi: 10.2337/diab.19.9.644 5469118

[24] Amendolara A Magoffin WD Naik AU Sant D Kriak J Green B . Chronic migraine may be associated with postprandial hypoglycemia in adult men: a case series. Cureus. (2024) 16:e54987. doi: 10.7759/cureus.54987 38550449 PMC10973797

[25] Jacome DE. Hypoglycemia rebound migraine. Headache. (2001) 41:895–8. doi: 10.1046/j.1526-4610.2001.01163.x 11703478

[26] Pearce J. Insulin induced hypoglycaemia in migraine. J Neurol Neurosurg Psychiatry. (1971) 34:154–6. doi: 10.1136/jnnp.34.2.154 5571601 PMC493726

[27] Levenson MA Levenson DI. Insulin as a potential treatment in selected migraine sufferers: two case reports. Ann Head Med. (2020) 2:30756. doi: 10.30756/ahmj.2020.02.08

[28] Lee S Choi S Kim HJ Chung Y-S Lee KW Lee HC . Cutoff values of surrogate measures of insulin resistance for metabolic syndrome in Korean non-diabetic adults. J Korean Med Sci. (2006) 21:695. doi: 10.3346/jkms.2006.21.4.695 16891815 PMC2729893

[29] Schrank Y Fontes R Perozo AFDF Araújo PB Pinheiro MFMC Gomes DMV . Proposal for fasting insulin and HOMA-IR reference intervals based on an extensive Brazilian laboratory database. Arch Endocrinol Metab. (2024) 68:e230483. doi: 10.20945/2359-4292-2023-0483 39529982 PMC11554367

[30] Tohidi M Ghasemi A Hadaegh F Derakhshan A Chary A Azizi F. Age- and sex-specific reference values for fasting serum insulin levels and insulin resistance/sensitivity indices in healthy Iranian adults: Tehran lipid and Glucose Study. Clin Biochem. (2014) 47:432–8. doi: 10.1016/j.clinbiochem.2014.02.007 24530467

[31] Hoffmann U Sukhotinsky I Eikermann-Haerter K Ayata C. Glucose modulation of spreading depression susceptibility. J Cereb Blood Flow Metab. (2013) 33:191–5. doi: 10.1038/jcbfm.2012.132 22968322 PMC3564186

[32] Wang Z Luo W Li P Qiu J Luo Q. Acute hyperglycemia compromises cerebral blood flow following cortical spreading depression in rats monitored by laser speckle imaging. J Biomed Opt. (2008) 13:064023. doi: 10.1117/1.3041710 19123669

